# DEAD-box RNA Helicase DDX3: Functional Properties and Development of DDX3 Inhibitors as Antiviral and Anticancer Drugs

**DOI:** 10.3390/molecules25041015

**Published:** 2020-02-24

**Authors:** Marina K. Kukhanova, Inna L. Karpenko, Alexander V. Ivanov

**Affiliations:** Center for Precision Genome Editing and Genetic Technologies for Biomedicine, Engelhardt Institute of Molecular Biology, Russian Academy of Sciences, Vavilov St. 32, 119991 Moscow, Russia; ilkzkil@gmail.com

**Keywords:** DEAD-box family RNA helicases, physico-chemical properties, antiviral drug, anticancer drug, inhibitors, virus life cycle

## Abstract

This short review is focused on enzymatic properties of human ATP-dependent RNA helicase DDX3 and the development of antiviral and anticancer drugs targeting cellular helicases. DDX3 belongs to the DEAD-box proteins, a large family of RNA helicases that participate in all aspects of cellular processes, such as cell cycle progression, apoptosis, innate immune response, viral replication, and tumorigenesis. DDX3 has a variety of functions in the life cycle of different viruses. DDX3 helicase is required to facilitate both the Rev-mediated export of unspliced/partially spliced human immunodeficiency virus (HIV) RNA from nucleus and Tat-dependent translation of viral genes. DDX3 silencing blocks the replication of HIV, HCV, and some other viruses. On the other hand, DDX displays antiviral effect against Dengue virus and hepatitis B virus through the stimulation of interferon beta production. The role of DDX3 in different types of cancer is rather controversial. DDX3 acts as an oncogene in one type of cancer, but demonstrates tumor suppressor properties in other types. The human DDX3 helicase is now considered as a new attractive target for the development of novel pharmaceutical drugs. The most interesting inhibitors of DDX3 helicase and the mechanisms of their actions as antiviral or anticancer drugs are discussed in this short review.

## 1. Introduction

Cellular proteins and cofactors have attracted much attention as new targets for the development of antiviral/anticancer drugs. Viruses are intracellular parasites that use host metabolic machinery for their replication and emission of infection [1]. More than 300 cellular proteins and co-factors participate in virus replication [2,3], but the most drugs approved by the Food and Drug Administration for the treatment of viral infections include drugs targeting viral enzymes. In the case of human immunodeficiency virus (HIV), the main targets are the reverse transcriptase, protease, and integrase, whereas only one drug (enfuvirtide, T-20, Fuseon) that blocks the cellular process of HIV fusion was approved for the treatment of HIV-infected people. For the treatment of hepatitis C virus (HCV)-infected patients, inhibitors of HCV NS3/4A protease, RNA-dependent RNA polymerase NS5B, and the nonstructural protein NS5A are used in clinics. The main problem with such an approach is the rapid development of viral resistance and escape of some genotypes/isolates from the action of the drugs. The use of host cell factors as targets for drug development can help to overcome the problem of viral resistance, since cellular proteins are much more conserved, and mutations in these proteins may alter cell viability [4,5,6]. The human DDX3 helicase, first identified in 1997 [7], is now considered as an attractive target for the development of novel pharmaceutical drugs [8,9,10,11]. The DDX3 helicase belongs to the large DEAD-box (Asp-Glu-Ala-Asp) family of ATP-dependent RNA helicases. The enzyme is a multifunctional protein implicated in all aspects of RNA metabolism, cell cycle regulation, and viral infection. The DDX3 helicase is involved in the replication of viruses belonging to different families: HCV [12], Dengue virus [13,14], Japanese encephalitis virus [15], and West Nile virus of the *Flaviviridae* family [16], HIV [17,18] of the *Retroviridae*, hepatitis B virus (HBV) [19] of *Hepadnaviridae*, Vaccinia virus of *Poxviridae* [20], Norovirus of *Caliciviridae* [21], influenza A virus of Orthomyxoviridae families [22], and several others. Knockdown of DDX3 blocks the replication of several types of viruses without essential toxic effects (for example, see [2,23,24]). DEAD-box helicases also attract a lot of attention as a target for the development of anticancer drugs, due to their role as oncogene in different types of tumors [25,26,27]. These data stimulated the synthesis of DDX3 inhibitors as antiviral/anticancer drugs. In order to design DDX3 selective inhibitors, a detailed knowledge of the substrate specificity of the enzyme, crystal structure, biochemical, and enzymatic properties of DDX3 is very essential.

## 2. Structure of the DDX3 Helicase and Its Enzymatic Properties

The general architecture of the DEAD-box helicase family is quite conservative. The helicases are composed of two RecA-like domains connected via a short flexible linker that allows changing their orientation to each other, which is critical for the enzyme functions [28,29,30]. Variable C- and N-terminals contain from a few to several hundred amino acids, which allow interaction with other proteins or RNA. RecA-like domains are composed of nine conservative motifs involved in ATP and RNA binding, ATP hydrolysis, and RNA strands unwinding.

**Figure 1:**
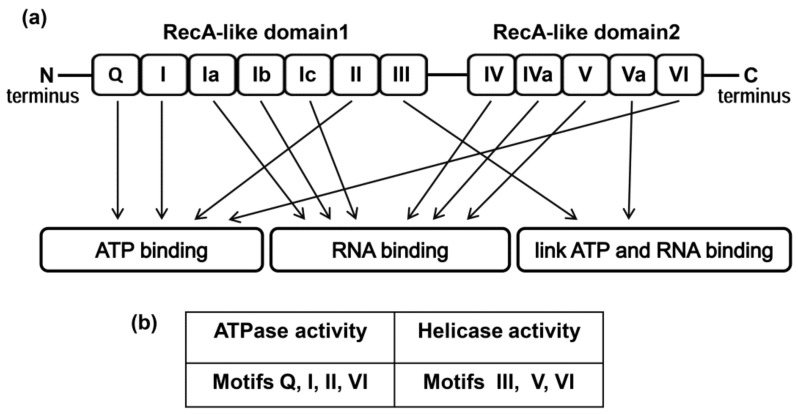
(**a**) Domain structure of the DDX3 helicase. (**b**) Motifs of RecA-like domains supporting ATPase and helicase activities.

The general domain structure of the DEAD-box family presented in Figure 1 is based on the study of crystal structures of the human DDX3 core [31,32] and its ortholog Drosophila Vasa [33], including their complexes with dsRNA, ADP, and a nonhydrolyzable ATP analogue. It was shown that the structure of the cores was typical for other members of the DEAD-box family, whereas the differences are mainly localized in the tail fragments [34]. It should be noted that the crystal structures of full-length DEAD-box helicases have not been solved yet.

The DDX3 core consists of two RecA-like domains (Figure 1). Both of them are comprised of several subdomains (motifs) [10,16,28,35]. Motifs Q, I, II, III, and VI are very important for both helicase and ATPase activities. The Q motif recognizes the adenine moiety of NTP, while motifs I and II bind the triphosphate moiety directly or through a Mg^2+^ ion, which was shown using a non-hydrolyzable ATP analogue, namely AMPPNP. As a result, ATP hydrolysis is performed by motif II. Motif V interacts with both the RNA and ATP. Motif III is associated with helicase activity. Motif V, together with Ia, Ib, and IV, is involved in RNA-binding and probably in helicase activity. Motif VI is responsible for the protein interaction with RNA during unwinding and ATP hydrolysis [35,36,37,38,39,40]. It is noteworthy that according to the crystal structure, interaction of the enzyme with single-stranded RNA leads to the formation of a closed form of the protein, thus promoting the formation of functional sites. The crystal structure of the DDX3 core model was extensively used to perform a virtual screening approach of different chemical compounds as potential inhibitors of the ATPase or helicase activities of DDX3 [40,41,42,43,44].

**Figure 2:**
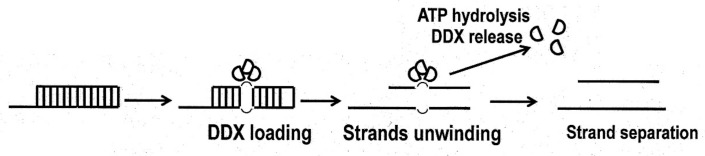
Local strand separation by DDX3 helicase trimer.

Several laboratories have intensively studied the substrate specificity of DDX3 ATPase/helicase for developing DDX3 inhibitors displaying antiviral/anticancer activities. In general, DDX3 helicase reveals biochemical features that are typical for RNA helicases, but this class of helicases has some specific functioning futures compared to processed helicases. In contrast to processed helicase, DDX3 helicase binds directly to oligonucleotide duplexes as an oligomer of two to three DDX3 molecules without translocation, and can completely separate short RNA-RNA or RNA-DNA duplexes using a single ATP molecule without its hydrolysis [36,45] (Figure 2).

This conclusion is strongly supported by the finding that the non-hydrolyzable ATP analog ADP-beryllium fluoride (ADP-BeF) can promote unwinding the short oligonucleotide duplex, although other non-hydrolyzable ATP analogs, ADP-aluminum fluoride (ADP-AlF4) and ADP-iminophosphate (ADPNHP), with structures similar to ATP do not provide RNA-RNA complex separation [46] but can form stable complexes with RNA [33,47]. Probably, minor differences in the structures of these compounds do not allow strand separation. As supposed, ATP hydrolysis is required for dissociation and recycling the DDX3 helicase, but not for the unwinding process (Figure 2). Such a mechanism fundamentally differs from that of processed helicases [33,46,48]. Substitution of the N7 nitrogen with carbon or the removal of the N6 amino group in the ATP molecule abrogates unwinding activity. These observations indicate the significance of the positions in nuclear bases for DDX3 functioning. There are some inconsistent data about substrate specificity of the DDX3 helicase. One series of publications showed that human DDX3 ATPase has wide substrate specificity and besides ATP, binds other rNTP, dNTP, as well as their L-stereomers [49]. It was also reported that ATPase activity is equally stimulated by the addition of DNA or RNA oligonucleotides. However, later publication presented the data that only ATP, but not dNTP or their analogs, are substrates of the DDX3 helicase. Moreover, ATPase activity is stimulated by DNA to a markedly lower level than that by RNA, and no significant ATPase activity in the absence of nucleic acid [45]. A virtual analysis of the interaction of the nuclear base and ribose residue of NTP with DDX3 crystal structure did not show any possibility of the relaxed substrate specificity [31]. The primer position in the double-strand RNA-RNA duplex is also important for the DDX helicase activity in vitro [33,45]. The enzyme shows a greater preference for the 3′-unpaired region of the duplex compared to those with 5’-unpaired regions and the inability to separate DNA-DNA complete duplexes. Fully double-stranded complexes DNA-RNA but not DNA-DNA can be separated, but much slower. 

## 3. Hypothetical Mechanisms of the DDX3 Helicase Role in Viral Replication

**Figure 3:**
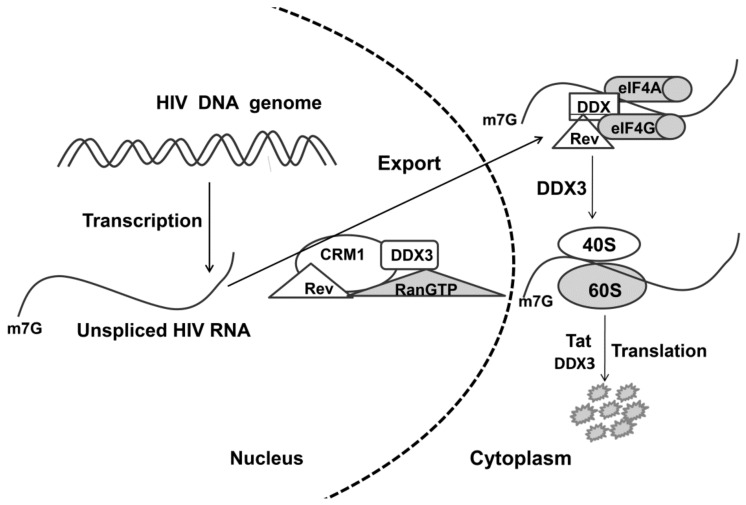
Hypothetical mechanism of involvement of the DDX3 helicase in the export of unspliced/partially spliced HIV RNA from nucleus to cytoplasm. CRM1—cellular export shuttle protein; Rev—shuttle protein with nuclear localization signal and a nuclear export signal. RANGTP—GTPase, Tat—HIV translation factor.

DDX3 helicase is a multifunctional protein interacting with many human and viral proteins and their complexes with RNA [17,48], but its role in the viral replication of different viruses has not been studied precisely. It was shown that DDX3 plays a dual function in viral replication: first, as a cofactor of viral replication, and second, as a mediator of the innate immunity system. The role of helicases in viral infection was discussed in several reviews [9,50,51]. Viruses recruit cellular helicases at different replication stages to overcome some rate-limiting stages in their replication. In this section, we describe the proposed mechanisms of action of the DDX helicases in the replication of HIV, HCV, HBV, herpes, and influenza viruses. The most detailed virus replication scheme involving DDX3 helicase has been described for HIV (Figure 3) [18,52,53,54,55]. DDX3 is a nucleo-cytoplasmic shuttling protein, which binds to the Rev/RRE/CRM1 transport complex for enhancing the export of unspliced/partially spliced HIV RNA from nucleus to cytoplasm. Knockdown of DDX3 using RNA interference or dominant-negative mutants suppresses Rev/RRE/CRM1 function in the export of full-length HIV RNA [17]. In addition to DDX3, other cellular RNA helicases, including DDX5, DDX17, DDX21, DHX36, DDX47, and DDX56, are involved in Rev-dependent nuclear export of HIV RNA [56].

Enhanced transport of RNA from the nucleus to the cytoplasm by the DDX3 helicase can be partly explained by unfolding of the HIV RNA secondary structure by the DDX3 helicase or shaking off associated with RNA proteins. DDX3 is also involved in HIV replication at the level of translation [57], and export from nucleus precedes the activation of translation through the loading of 43S preinitiation complex on 5′-UTR [53] and probably by overall promotion of 80S ribosome assembly [58]. Moreover, this hypothesis is based on the specific association of DDX3 with HIV translation factor Tat, which facilitates Tat-dependent translation of viral genes [55,59].

In contrast to DDX3 functions facilitating viral mRNA transport and translation, DDX3 participates in the anti-viral innate immune signaling pathway, leading to type I IFN induction after phosphorylation by TBK1/IKKε and translocation into the nucleus, leading to the activation of the IFNβ promoter [60,61,62]. The viruses, in turn, try to overcome the host immune system, targeting DDX3.

Much more data exist for HCV infection, but DDX3’s role in its replication is less clear. Indeed, the DDX3 helicase is also an essential component in HCV replication [63]. It was reported that siRNA-mediated knockdown of DDX3 causes a reduction in HCV RNA and HCV core expression levels in cells. The first data about the interaction of DDX helicase with C-terminal domain of HCV core were published in 1999 [64,65,66]. The authors hypothesized that the interaction of DDX3 with HCV genotype 1 core somehow increased HCV replication level. However, later, the statement was overcome; namely, mutations in DDX3 genotype 2a, which prevented the binding of DDX3 to the HCV core, had no effect on HCV replication, although core-derived peptides of HCV genotype 1b inhibited HCV replication [67]. These contradicted results can be explained by the different type of HCV genotypes used by the authors [68]. Later, Oshiumi et al. explained that HCV core inactivates the IPS-1 adaptor, triggering RIG-mediated IFN-beta induction caused by DDX3 and, as a result, virus replication is propagated [69,70]. So, the authors concluded that DDX3 is a RigI adaptor protein. However, this mechanism is not quite clear, as many cell lines used in HCV research are not competent for IFNβ induction. For example, the most common cell line, Huh7.5, is characterized by inactivation of Rig-I (DDX58) [71]. In addition, HCV NS3 protease triggers proteolysis of MAVS, a classical Rig-I adaptor critical for interferon induction [72]. However, recently, Horner’s group described a novel TRF3-dependent but Rig-I/MAVS-independent interferon response controlled by HCV [73]. So, the input of interferon signaling in the control of HCV infection and the precise role of DDX3 in its modulation still have to be investigated. 

An alternative mechanism by which DDX3 facilitates HCV replication could be an alteration of lipid droplet (LD) biogenesis and homeostasis [67,74]. Lipid droplets are the ER-derived organelles on which several viral proteins, including HCV core, are localized. Moreover, the initial step of virion assembly, namely HCV encapsulation into nucleocapsids, occurs at the LD-ER interphase, and HCV core and NS5A control this process [75,76]. LD biogenesis is induced by the interaction of DDX3 with 5′-UTR of the HCV RNA, which leads to relocalization of IKKα and concomitant SREBP activation [74]. HCV core also triggers relocalization of DDX3 to LDs, although direct core-DDX3 interaction is not required for virus replication or virion production [67]. It is tempting to speculate that DDX3 could also affect the initiation of replication or nucleocapsid formation via affecting NS5A, whose phosphorylation affects the interaction of viral RNA with RNA-dependent RNA polymerase [77] or HCV core [78]. DDX3 is also a regulator of casein kinase 1ε [79], one of the kinases that drives NS5A hyperphosphorylation [80]. Hyperphosphorylation of the NS5A protein is considered one of the factors that promotes transition from replication to virion assembly [81]. However, DDX3 silencing does not affect the ratio between the hyperphosphorylated (p58) and basally-phosphorylated (p56) forms of the protein [82], so this interaction probably does not affect replication of the virus.

Finally, DDX proviral action could be due to its role in the regulation of stress granules (SG) and processing bodies (P-bodies). SG are membraneless organelles that consist of proteins with distorted structure and various RNAs. Specifically, most mRNAs sequestered to SGs are under stalled translation, so the formation of these organelles suppresses global and gene-specific translation, and in particular, may result in changes in interferon response [83]. In P-bodies mRNAs are stored for decapping and degradation or until initiation of translation. However, the biology of stress granules and P-bodies is not well understood, so the role of DDX3 in their functioning merits further studies. DDX3 is a regulator of SG assembly [84] and maturation [85]. The assembly of stress granules is enhanced in HCV-infected cells [86]. The binding of DDX3 to 5′-UTR of HCV RNA promotes interaction of the latter with SGs as well as with lipid droplets [87]. Noteworthy, SG assembly is inhibited in tumor cells harboring DDX3 with tumor-associated mutations [88], i.e., the inactivated protein [89]. So, DDX3 inhibitors could be regarded as tools to prevent virus-induced formation of SGs. The formation of stress granules may affect HCV replication by a global change in host gene translation. Indeed, DDX3 binds to a vast majority of mRNAs, as well as to 18S rRNA, thus blocking their translation [90]. However, as shown for yeast DDX3 orthologue Ded1, the helicase is also critical for reinitiation for translation [91]. Specifically, DDX3-mediated SG assembly may significantly affect the translation of mRNAs bearing upstream open reading frames (uORFs) [92,93] and probably cap-independent translation. Indeed, for enterovirus serotype 71, which is also a small enveloped RNA virus, DDX3 does stimulate IRES-mediated translation of its genome [94]. Another recent paper suggested that DDX3 is also a regulator of non-AUG initiated translation [95]. So, the role of DDX3 in virus replication can also be explained by a switch from cap-dependent (i.e., cellular) to cap-independent (i.e., viral) RNA translation. However, it is evident that investigation of stress granule biology may unveil their role in the replication of viruses and in controlling innate immune response.

Similar events occur for other members of the *Flaviviridae* family and for Picornaviruses. DDX3 interacts with UTRs of the Japanese Encephalitis virus and with its NS3 and NS5A proteins also to promote late stages of the viral life cycle [15]. The West Nile virus sequesters DDX3 from P-bodies to sites of viral replication, thus affecting the functioning of these organelles but promoting its own replication [96]. So, the mechanisms by which DDX3 promotes the replication of HCV could probably be expanded to these viruses as well.

In contrast to the proviral effect of DDX towards HIV and HCV, DDX3 restricts HBV replication [97]. There is very little information on the mechanism for suppressing HBV genome replication by the DDX3 helicase. The first suggestion assumed that DDX3 inhibits virus replication by interacting with HBV DNA polymerase after DDX3 encapsulation, thus preventing the transcription stage [19]. However, later, it was shown that the level of virus was independent of the interaction of HBV polymerase with DDX3 [97]. The second hypothesis assumed that DDX3 helicase competes with HBV DNA polymerase for the interaction with transcription factor TBK1/IKK(epsilon) blocking the interferon induction [98]. DDX3 overexpression could prevent the inhibitory effect of HBV replication. This assumption is consistent with the stimulation of the immune system by the over expression of DDX3 helicase [99].

Interestingly, HBV is not the only virus whose virions contain DDX3. Another example is herpes simplex virus type 1 (HSV-1) [100]. However, in this case, DDX3 acts not as an antiviral but a proviral factor [101], presumably by affecting the expression of viral genes and regulating virion assembly [102]. This could be a feature of various herpes viruses, as similar data point to DDX3 as a proviral gene for the replication of human cytomegalovirus, which also belongs to the *Herpesviridae* family [103]. However, the mechanisms of their participation do not involve the regulation of interferon production and concomitant signaling, whereas in HCMV-infected cells it does enhance the production of IFNβ [104].

In the case of the influenza A virus (IAV), DDX3 acts as an antiviral factor [22]. It interacts with NS1 and NP proteins [22]. DDX3 is also recruited to viral replicase [105], presumably by interaction with PB1-F2 (a subunit of viral replicase) [106]. In the case of the latter, such interaction is significantly enhanced in the case of a highly pathogenic 1918 strain, and results in a co-degradation of both DDX3 and viral protein [106]. So, it could be one of the factors of an extremely high pathogenicity of this viral strain. Another factor is an NS1- and NP-induced DDX3-mediated formation of SGs [22]. The third one could be a DDX3-mediated induction of IFNβ through stimulator of interferon genes (STING) [107]—another cytoplasmic sensor of viral nucleic acids. However, contrary data also exist. Diot et al. considered DDX3 as a proviral factor [108]. In this study, DEAD-box helicases were regarded as factors that promoted export from the nucleus to the cytoplasm of influenza A virus mRNA. Replication of IAV was significantly impaired upon the silencing of 14 of 35 studied DEAD-box proteins, with DDX19 helicase being the most effective in reducing infectious IAV [108].

Finally, DDX3 may be a cellular factor that also promotes the development of virus-associated pathologies. As discussed above, the HCV core via DDX3 promotes the formation of lipid droplets [74], thus contributing to the development of liver steatosis, one of the common liver diseases in chronic hepatitis C patients. An additional link between HCV-induced changes in lipid homeostasis and DDX3 is provided by the downregulation of microsomal triglyceride transfer protein (MTP), achieved via HCV-induced suppression of DDX3 expression [109]. The DDX3 helicase also regulates the production of proinflammatory cytokines and chemokines by several mechanisms. The expression of cytokines is controlled by the NFkB factor, whereas their subsequent maturation by the NLRP3 inflammasomes. DDX3 interacts with NFkB and suppresses its activity [110]. Activation of inflammasomes is suppressed during sequestration of DDX3 to stress granules [111]. So, DDX3 could affect the development of inflammation during various infections, including HCV, HIV, and respiratory viruses [112,113,114]. Indeed, in the case of HIV, DDX3 also promotes Tat-associated neurotoxicity, and since it is one of the best studied inhibitors, the compound RK-33 suppresses the production of proinflammatory cytokines induced by Tat [115].

## 4. Inhibitors of the DDX3 Helicase

During the last 15 years hundreds of chemical compounds of different classes have been elaborated as potential inhibitors of ATPase/helicase of DDX3 helicase [41,42,43,44,116,117]. Herein, we focus our attention only on the most active and nontoxic compounds inhibiting DDX3 helicase as well as blocking virus replication or suppressing tumor progression.

The first potential DDX3 inhibitors were synthesized after a high-throughput docking prediction of the interaction of commercially available compounds from the Asinex Co. (Asinex Ltd., Winston-Salem, NC, USA, http://www.asinex.com) and ChemBridge Co. (ChemBridge Corporation USA, San Diego, CA, USA (http://www.chembridge.com)) with the crystal structure of the DDX3–AMP complex in closed conformation [31,118]. Later on, their structures were optimized and the compounds were tested as inhibitors of DDX3 in vitro and some of them as suppressors of virus replication in cell cultures and tumor aggression. Among the active compounds were derivatives of rhodanine, pyrazolo-, or diarylurea [2] as well as triazine-, naphthyl-, pyrazolo-, ring-expanded nucleoside, and others [42,43,116,117,118,119].

Several compounds among rodanine and triazine derivatives were discovered as inhibitors of ATPase activity of DDX3 at low micromolar concentration in vitro, but their anti-HIV activities in cell cultures were one order of magnitude higher compared with the cell-free system. Moreover, the compounds displayed rather high cytotoxicity [2,117]. Further investigations of these derivatives were not undertaken. The interesting compounds were discovered among a series of diarylurea derivatives (Table 1) [8].

The compounds **A** and **B** inhibited DDX3 helicase activity with IC_50_ 1 μM and 6 μM, respectively, being a competitive inhibitor with respect to RNA substrate [41]. Taking **A** and **B** as leading compounds, the compounds were modified after docking analysis of the crystal structure of the complex DDX3 + compound, using the 3D structure of DDX3 in its closed conformation [31]. The most interesting among the predicted inhibitors was the compound **1**, bearing a triazole ring instead of the nitro group. The compound inhibited DDX helicase activity with IC_50_ 0.3 μM and displayed inhibiting potential against HIV, HCV, Dengue virus, and West Nile virus replication in cell cultures, with ED_50_ 1.1 μM for HIV, 0.97 μM for HCV, 2.55 μM for Dengue virus, and suppressed West Nile virus replication by 98% at a concentration of 20 μM. The cytotoxicities of the compounds were rather low (>200, 50, 200 µM, respectively) [8,16]. Importantly, the compounds also suppressed HIV1 strains resistant to anti-HIV drugs. One of the essential disadvantages of the compounds was low aqueous solubility, which makes it difficult to study the inhibitors in animal models. 

After further virtual screening and modeling of the interaction of DDX helicase with the Sinex library of compounds, several potential DDX inhibitors were synthesized and tested as inhibitors of DDX helicase activity. The experimental results confirmed that **4** and **5** (Table 2) among the 27 selected compounds inhibited the DDX3 helicase activity with high potency (IC_50_ 0.2–0.3 μM) [118].

Based on compound **5** and previously published compound **2** (Table 1), a new series of DDX3X inhibitors was synthesized, validated as inhibitors of both DDX3 helicase in vitro and the WNV replication [16]. The most interesting compound proved to be compound **6**, which showed high activity against WNV and a good safety profile. The docking studies showed that the compound acts as a competitive inhibitor towards a template in the helicase binding site. Compound **6** displayed the highest antiviral activity among the series, inhibiting WNV replication by 98% at 20 μM.

**Figure 4:**
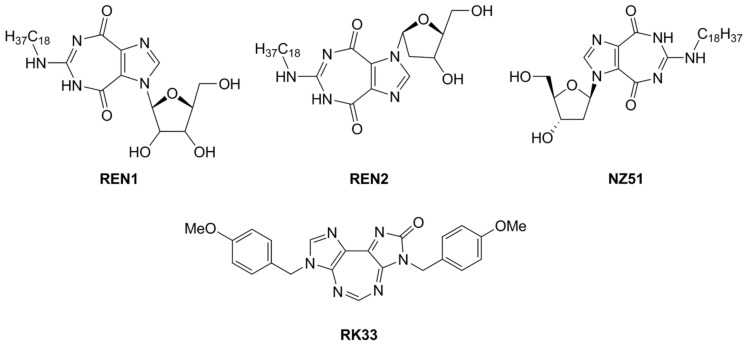
DDX3 inhibitors with anticancer activity.

Another interesting class of DEAD-box cellular helicase inhibitors is presented by ring-expanded nucleosides containing imidazo [4,5-e][1,3]diazepine ring or imidazo [4,5-e]]1,2,4 triazepine ring systems (RENs). The compounds strongly inhibit human DDX3 helicase, HCV viral helicases (NS3 helicase), helicases of West Nile virus, and Japanese encephalitis virus in a cell-free system, and, as a result, virus replication is blocked in the concentration range 5–15 µM [43,44,119,120,121,122]. The most effective inhibitors of human DDX3 among the REN class of the compounds are presented in Figure 4.

The observation of the simultaneous inhibition of HIV and HCV by REN1 and REN2 is very important, since HCV is a frequent co-infection in AIDS patients, leading to liver cirrhosis and death. As supposed, the compounds mimic nucleoside, occupy the ATP binding site of human or viral helicases and delay or interrupt virus replication. The toxicity of the compounds was observed neither in cell cultures nor in a mice model [119].

Later on, it was shown that RENs display not only antiviral but also anticancer activities. The role of DDX3 in cancer development is rather controversial. DDX3 helicase can act as an oncogene or tumor suppressor in different cancer types [26,27,116,121,122,123,124,125]. Moreover, DDX3 may play different roles in the same type of cancer. For example, a decreased level of DDX3 was found in hepatocellular carcinoma (HCC) caused by HBV, although not by HCV [126]. DDX3 also plays dual roles in breast cancer [127] and colorectal cancer patients [128,129]. Up to now, there is no exact explanation of the dual role of DDX in a variety of cancers, but some data should be taken into consideration. DDX3 is involved in the cell signaling pathway Wnt/β-catenin and can affect the Wnt regulation cascade, which is crucial to DDX3 functions in cancer development [130]. DDX3 also modulates cell adhesion, represses the E-cadherin expression, which results in increased cell migration, and thus promotes tumor progression [131]. As supposed, different roles of DDX helicases might be associated with mutations in the DDX helicase (as can be exemplified by [132]) or virus infections, particularly HCV or HBV. DDX3 knockdown with short interfering RNA (shRNA) or small molecules suppressed cell motility and reduced metastatic potential in cancer cells and a mouse model [43,131]. The localization of DDX3 within the cell might also determine different DDX3 functions. Usually, DDX3 accumulates in the cytoplasm of the cell, but there are also reports of DDX3 export from nucleus to cytoplasm during tumor progression. DDX3 helicase is a nucleo-cytoplasmic shuttling protein predominantly localized in the cytoplasm of non-malignant cells. It has been suggested that its localization is altered during cell transformation and could even contribute to malignancy [133,134].

Among synthesized REN analogs (Figure 4), NZ51 suppresses ATPase/helicase of DDX3 at low micromole concentration in vitro and displays antiproliferative activity, blocking cell replication at the G1 phase of aggressive breast cancer in different cell cultures [43], causing a global delay in cell cycle progression [135]. The observed effects were similar to those upon the silencing of the DDX3 gene. Unfortunately, NZ51 treatment had no effect on primary tumor growth rates in a mouse model system, although DDX3 knockdown by shRNA resulted in reduced tumor volume and metastasis progression [43]. Among ring-expanded derivatives, the compound RK33 proved to be the most interesting and prospective for medicine (Figure 4). RK-33 was found to display antiproliferative activity against Ewing sarcoma [136], breast cancer [137], medulloblastoma [25], colorectal [129], prostate [138], and lung [116] cancer, due to a stage G1 arrest [43]. Moreover RK-33 proved to be a radiosensitizer that allows a reduction in the dose of radiation for cancer treatment [45]. The precise mechanism of RK-33 action in different types of cancer remains to be elucidated. There is evidence that the inhibition of DDX3 functions by RK-33 could disrupt the DDX3-β-catenin complex and cause a disturbance in the Wnt signaling pathway involved in cell differentiation, cell proliferation, malignant tumors, and transition of the G1/S cell cycle [131].

## 5. Conclusions

DDX3 is considered a potential new chemotherapeutic target for the treatment of viral infections and different types of cancer. There are about 50 human DEAD-box helicases involved in a variety of cellular and viral metabolic processes. In spite of the fact that interfering in cellular integrity poses a risk of toxic effects, some effective chemical inhibitors of DDX3 enzymatic activity have been developed, which suppress viral replication in cell cultures and display anticancer activity against a number of cancer types without significant toxicity. One of the inhibitors of DDX3, ATPase RK-33, was recommended for the preclinical stage against lung cancer. Moreover, RK33 increases radiosensitivity, which allows a reduction in the dose of radiation for cancer treatment. Little is currently known about the mechanisms for suppressing viral infections and regression of tumors with DDX helicase inhibitors. Sometimes published data are contradictory, which is probably due to different testing systems. To design new effective DDX inhibitors much work remains to be done to unveil the mechanism of suppression of virus replication and tumor repression. However, the results obtained allow us to hope that the chosen direction is correct and in the future will give new effective therapeutic drugs.

## Figures and Tables

**Table 1:** Selected diarylurea derivatives as DDX3 inhibitors in vitro.

General Structure	
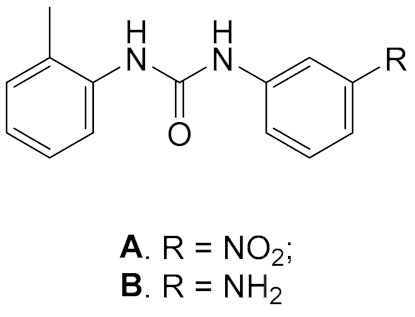	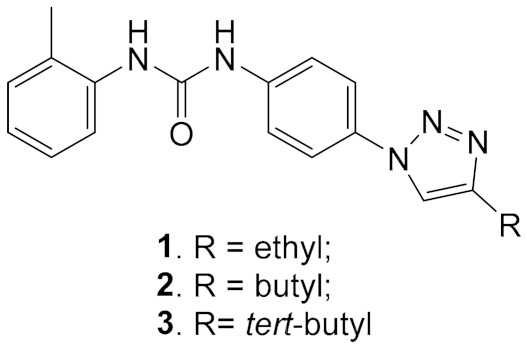
A. IC_50_ 1 μM; B. IC_50_ 6 μM	1. IC_50_ 0.3 μM; 2. IC_50_ 0.98 μM; 3. IC_50_ 3.36 μM

**Table 2:** Inhibitors of DDX3 helicase activity.

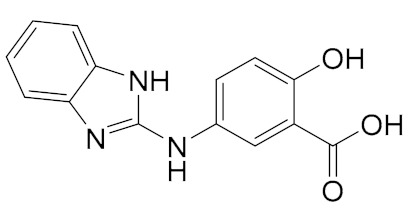	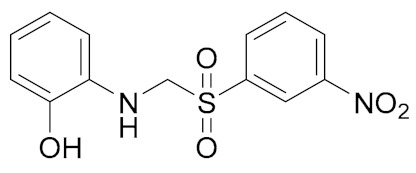	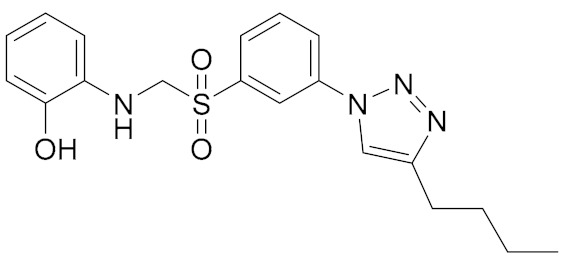
4. IC_50_ 0.2 μM	5. IC_50_ 0.3 μM	6. IC_50_ 0.3 μM
